# Supplementary material for: Artificial diet alters activity and rest patterns in the olive fruit fly
Source: PLoS One. 2023 Feb 21;18(2):e0274586. doi: 10.1371/journal.pone.0274586 (PMC9943008; doi:10.1371/journal.pone.0274586)
Supplement: Attachment — Submitted filename: PlosOne D-22-24097_Reviewer.docx [file pone.0274586.s006.docx]

Manuscript number: PONE-D-22-24097

Title: Laboratory rearing alters activity and sleep patterns in the olive fruit fly

Point to point comments related to the manuscript, indicating where changes are necessary or recommended

| **Line number** | **Suggestions, comments or questions** |
| --- | --- |
| **Title** | |
| 1 | The title is concise but I think it might be more precise specifying “Artificial diet alters activity and …” instead of “Laboratory rearing alters activity and…” since only the diet differs, not the lab conditions. |
| **Abstract** | |
| 25 | Are the words “that are” necessary? I would delete them. |
| 26 | I suggest to substitute “for this pest” with “against this pest” |
| 27 | Substitute “their” with “its” (the quality of the colony) or change the sentence into “However, the colony’s laboratory adaptation can affect the quality of the reared flies.” |
| 27-28 | Usually equipment details are not required in the abstract. Consider to delete the sentence within parentheses “(LAM25, Trikinetics, 28 MA, USA)”. |
| 35 | I think the word “females” should be singular, that is “female” |
| **Introduction** | |
| 47 | To support more strenghtly the sentence I suggest to cite also the article DOI: 10.1111/j.1365-294X.2005.02610.x, although a bit older, respect to the cited article [3] that deals with exploration for olive fruit fly parasitoids. |
| 78-80 | I think this sentence, although formally correct, should be moved to Materials and Methods chapter since it states a methodological parameter used in the current research. |
| **Materials and methods** | |
| 93 | I think you can omit “colony”. |
| 94 | Approximately, how many flies/cage? (see also below) |
| 95 | It seems the same diet was used to feed AR adults (see lines 104-195). If I understood well, please, put forward the sentence “in the form of a liquid diet consisting of…” to the line 95 and in line 104 specify that adults were fed with the same liquid diet. |
| 96 | Was it a cotton wick or just a cotton wad? |
| 104 | Here is specified that the cage contained about 200 flies, please, specify also the approximate number of wild flies/cage at line 94 (see comment at line 94). |
| 111 | In order to make the rearing of olive fruit fly on the artificial larval diet replicable by other scientists, I think you should specify the company names of some critical ingredients, such as brewer’s yeast, soy hydrolysate and cellulose. I am asking that because most of the providers reported by Tsitsipis 1977 are not available any more. |
| 117-118 | Was the diet again in a liquid form? Please, specify that. |
| 92-127 | I understand that W and AR flies have been kept under the same conditions, however it is known that *B. oleae* is a species demanding high light intensity and this can affect its behavior. Can you provide details on the light intensity supplied to the cages? |
| 131 | Please, after Trikinetics add details about town and country of the company, i.e. “(Trikinetics Inc, Waltham, MA, USA)” |
| 143-144 | I see that this automatic device allows to record much and detailed data simultaneously. However, you studied small groups of flies (from 12-14 up to 32 specimens) each of them obtained from just one cohort. I wonder if you could have obtained more variable data studying flies from different cohorts. Can you provide citations to support the robustness of your choice or there are evidences of intra-group variability highlighted by this kind of study, for instance in Drosophila? |
| **Results** | |
| 165-274 | In the whole chapter Results (and figure captions as well) mean values are shown followed by “(SE)” without the “±” symbol, moreover standard error values are shown within parentheses after mean numbers. Maybe I am wrong and you use a different syntax I am not familiar with, however I think this way of showing data can confuse the reader. I explain how to show numbers in an example in the following comment. |
| 166-167 | I think the sentence should be written as following: “The mean locomotor activity level (±SE) of W virgin males during the LP was 2031.3 ±132.0 counts and that of W virgin females was 1394.2 ± 86.6 counts.” Did you mean this? If yes, please, insert the symbol “±” throughout all the Results chapter and figure captions, before SE values. |
| 166 | Delete “was”. |
| 185 | Add “during LP” at the end of the sentence, after “females”. |
| 187-190 | Please, specify that locomotor activity during DP is not shown in Fig 2. |
| 273-274 | In this figure caption you specified colors used in the graph for the two categories. It helps in understanding data, however I suggest to change the series names to avoid confusion: substitute “Light period” with “rest episodes” and “Dark period” into “sleep episodes”. Increase also the font size of all the characters except the Y axis. |
| **Discussion** | |
| 277-278 | I think this sentence is a bit imprecise since some factors affecting the locomotor activity are clearly defined in the two options, while other are not. To be more consistent, what about writing “This study indicates that artificial rearing, mating status and photoperiod phase affect the locomotor activity of the olive fruit fly adults.” |
| 279 | Change “during this period” into “during this phase” |
| 282 | Change “in accordance to [34].” into “in accordance to past studies [34].” |
| 286 | I do not understand what you mean: how monogamy could cause lower locomotor levels? |
| 289 | When you write “due to oviposition behavior”, do you mean that oviposition might consume energy so that females need longer rest? If yes, write it down more clearly. |
| 289-300 | Although present findings are compared, discussed and supported by relevant literature, I think that the huge work by Economopoulos has to be acknowledged and taken into consideration. He studied the mating behavior of wild and artificially reared olive fruit flies, evidencing some differences highlighted also by your research, for instance the earlier mating time of the AR males (without mentioning genetic changes between W and AR flies). I am suggesting only some articles that should be discussed: <https://doi.org/10.1093/ee/11.1.17>; Economopoulos, A. P., Zervas, G. A., 1982. The quality problem in olive flies produced for SIT experiments. In: *Sterile insect technique and radiation in insect control*. International Symposium on the Sterile Insect Technique and the use of Radiation in Genetic Insect Control, IAEA SM-255/39: 357-368. |
| 301 | Delete “of”. |
| 332 | It seems this sentence refers to the article [50], so, please, insert “[50]” at the end of the sentence. |
| 337 | Before or after this last paragraph I suggest to recall what stated in the last sentence of the abstract “We discuss how locomotor activity and sleep pattern differences may affect the laboratory flies’ ability to compete with wild males in the field.” You should relate your findings to the importance of producing high quality mass reared insects for SIT purposes. |
| **References** | |
| 31 | Amend “Kouloussis” instead of “Koulousis” |
| 40 | Write “Coquillet” instead of “COQUILLET” |
| **Figures** | |
| Figure 1 | Please, increase the font size of the legend (W virgin male etc.) |
| Figure 2 | Please, increase the font size of all the characters. |
| Figure 3 | Please, increase the font size of the legend (AR virgin male etc.) |
| Figure 5 | Please, increase the font size of all the characters except the Y axis |
| Figure 6 | I suggest to maintain the same pale and dark gray colours used in previous figures. Increase also the font size of all the characters except the Y axis. |
