# Supplementary material for: Artificial diet alters activity and rest patterns in the olive fruit fly
Source: PLoS One. 2023 Feb 21;18(2):e0274586. doi: 10.1371/journal.pone.0274586 (PMC9943008; doi:10.1371/journal.pone.0274586)
Supplement: Attachment — Submitted filename: Response to Reviewers.docx [file pone.0274586.s007.docx]

**Response to the Editor comments:**

Journal Requirements:

When submitting your revision, we need you to address these additional requirements.

1. Please ensure that your manuscript meets PLOS ONE's style requirements, including those for file naming. The PLOS ONE style templates can be found at 
<https://journals.plos.org/plosone/s/file?id=wjVg/PLOSOne_formatting_sample_main_body.pdf> and 
<https://journals.plos.org/plosone/s/file?id=ba62/PLOSOne_formatting_sample_title_authors_affiliations.pdf>

**Response 1. We updated the revised MS according to the style requirements. Figure titles that were not included previously are added. The references style was also adjusted.**

2. We noticed you have some minor occurrence of overlapping text with the following previous publication(s), which needs to be addressed:

- <https://www.frontiersin.org/articles/10.3389/fnins.2019.01271/full> [48]

**Response 2. The phrase has been re-written with an additional reference to support it (lines 355-356 of unmarked MS): In drosophila flies, the loss of sleep can have a negative impact in their brain function [19], although the sleep deprivation effects in adults can be reversed through recovery sleep [46, 47].**

- <https://pubmed.ncbi.nlm.nih.gov/11854509/>	[50]

**Response 2. The text and reference have been removed, after we re-wrote the discussion section accordingly to reviewers’ suggestions**.

- <https://faseb.onlinelibrary.wiley.com/doi/epdf/10.1096/fj.202001107R>	[44]

**Response 2. The text and reference have been removed, after we re-wrote the discussion section accordingly to reviewers’ suggestions**.

In your revision ensure you cite all your sources (including your own works), and quote or rephrase any duplicated text outside the methods section. Further consideration is dependent on these concerns being addressed.

3. In your Methods section, please provide additional information regarding the permits you obtained for the work. Please ensure you have included the full name of the authority that approved the field site access and, if no permits were required, a brief statement explaining why.

**Response 3. No field access permit was needed, as infested olive fruits were not collected from a commercial grove, but from abandoned or ornamental trees (line number 85-87 of the unmarked MS)**

4. We note that the grant information you provided in the ‘Funding Information’ and ‘Financial Disclosure’ sections do not match.

When you resubmit, please ensure that you provide the correct grant numbers for the awards you received for your study in the ‘Funding Information’ section.

**Response 4. We added the scholarship details received by AMT from the State Scholarship Foundation in the Funding Information section. The correct Financial disclosure is also given in the latest cover letter.**

5. In your Data Availability statement, you have not specified where the minimal data set underlying the results described in your manuscript can be found. PLOS defines a study's minimal data set as the underlying data used to reach the conclusions drawn in the manuscript and any additional data required to replicate the reported study findings in their entirety. All PLOS journals require that the minimal data set be made fully available. For more information about our data policy, please see <http://journals.plos.org/plosone/s/data-availability>.

Upon re-submitting your revised manuscript, please upload your study’s minimal underlying data set as either Supporting Information files or to a stable, public repository and include the relevant URLs, DOIs, or accession numbers within your revised cover letter. For a list of acceptable repositories, please see <http://journals.plos.org/plosone/s/data-availability#loc-recommended-repositories>. Any potentially identifying patient information must be fully anonymized.

Important: If there are ethical or legal restrictions to sharing your data publicly, please explain these restrictions in detail. Please see our guidelines for more information on what we consider unacceptable restrictions to publicly sharing data: <http://journals.plos.org/plosone/s/data-availability#loc-unacceptable-data-access-restrictions>. Note that it is not acceptable for the authors to be the sole named individuals responsible for ensuring data access.

We will update your Data Availability statement to reflect the information you provide in your cover letter.

**Response 5. We have uploaded the dataset at zenodo repository.** <https://doi.org/10.5281/zenodo.7221901>

**Response to Reviewer’s #1 comments:**

Reviewer #1: The manuscript contains two major problems. First the inactivity is not clearly defined as sleep or what kind of sleep. Fatigue, lethargus, or true sleep? Is this inactivity accompanied with circadian timing, fixed sleep site, fixed sleep posture, rebound from the deprivation of sleep or fixed encephalographic patterns? Second, the change is associated with any circadian parameters like phase, amplitude, period, synchrony etc. Is the change associated with any circadian gene expression dynamics? We cannot make further scientific analysis from this point. Current condition remains incomplete condition.

**Response: We agree that the term sleep has to be defined by the parameters that the reviewer has listed. We have conducted an additional bioassay with wild olive fruit flies to observe the preferred body posture and position in the tubes were the flies are maintained. Photos and videos are provided as supporting information.**

**Encephalographic patterns and gene expression research cannot to be conducted in our laboratory. Further bioassays with laboratory reared flies were not possible, as we no longer maintain the specific colony in our laboratory.**

**Since we cannot define the episodes of inactivity as sleep episodes, we used the term “rest” throughout the text. The discussion was re-written to support more strongly the importance of quality laboratory reared flies for the SIT, as suggested by the other reviewer. The parts of the discussion referring to the sleep state of flies were removed.**

**Response to Reviewer’s #2 comments:**

Point to point response to comments

| **Original MS Line number** | | | **Revised MS (unmarked version) Line number** | | **Suggestions, comments or questions + responses** |
| --- | --- | --- | --- | --- | --- |
| **Title** | | | | | |
| 1 | | **1** | | The title is concise but I think it might be more precise specifying “Artificial diet alters activity and …” instead of “Laboratory rearing alters activity and…” since only the diet differs, not the lab conditions.  **Response: The title was altered to “Artificial diet affects activity and rest patterns of the olive fruit fly”** | |
| **Abstract** | | | | | |
| 25 | **21** | | | Are the words “that are” necessary? I would delete them.  **Response: They are deleted.** | |
| 26 | **22** | | | I suggest to substitute “for this pest” with “against this pest”  **Response: It is substituted** | |
| 27 | **23** | | | Substitute “their” with “its” (the quality of the colony) or change the sentence into “However, the colony’s laboratory adaptation can affect the quality of the reared flies.”  **Response: The suggested phrase has been added** | |
| 27-28 | **23** | | | Usually equipment details are not required in the abstract. Consider to delete the sentence within parentheses “(LAM25, Trikinetics, 28 MA, USA)”.  **Response: it is deleted** | |
| 35 | **31** | | | I think the word “females” should be singular, that is “female”  **Response: it is corrected** | |
| **Introduction** | | | | | |
| 47 | **41-42 and reference 4** | | | To support more strenghtly the sentence I suggest to cite also the article DOI: 10.1111/j.1365-294X.2005.02610.x, although a bit older, respect to the cited article [3] that deals with exploration for olive fruit fly parasitoids.  **Response: The reference was added and the sentence improved to contain the geographical distribution of the olive fruit fly** | |
| 78-80 | **164-166** | | | I think this sentence, although formally correct, should be moved to Materials and Methods chapter since it states a methodological parameter used in the current research.  **Response: It is moved to lines 164-166 of the revised (unmarked version) of the MS** | |
| **Materials and methods** | | | | | |
| 93 | **87** | | | I think you can omit “colony”.  **Response: it is omitted** | |
| 94 | **88** | | | Approximately, how many flies/cage? (see also below)  **Response: approximately the same density as the AR flies, 180-200 per cage.** | |
| 95 | **91** | | | It seems the same diet was used to feed AR adults (see lines 104-195). If I understood well, please, put forward the sentence “in the form of a liquid diet consisting of…” to the line 95 and in line 104 specify that adults were fed with the same liquid diet.  **Response: It is the same liquid diet and it is now specified in line 91 of the revised MS** | |
| 96 | **93** | | | Was it a cotton wick or just a cotton wad?  **Response: It was a cotton wick.** | |
| 104 | **88** | | | Here is specified that the cage contained about 200 flies, please, specify also the approximate number of wild flies/cage at line 94 (see comment at line 94).  **Response: The number was specified in line 88 of the revised MS** | |
| 111 | **107-111** | | | In order to make the rearing of olive fruit fly on the artificial larval diet replicable by other scientists, I think you should specify the company names of some critical ingredients, such as brewer’s yeast, soy hydrolysate and cellulose. I am asking that because most of the providers reported by Tsitsipis 1977 are not available any more.  **Response:** **The commercial names of the used ingredients are mentioned in lines 107-111** | |
| 117-118 | **117-118** | | | Was the diet again in a liquid form? Please, specify that.  **Response: It was the same liquid diet, as specified now in lines 117-8** | |
| 92-127 | **89-91** | | | I understand that W and AR flies have been kept under the same conditions, however it is known that *B. oleae* is a species demanding high light intensity and this can affect its behavior. Can you provide details on the light intensity supplied to the cages?  **Response: Light intensity and photometer used for measurement are mentioned in lines 89-91** | |
| 131 | **131** | | | Please, after Trikinetics add details about town and country of the company, i.e. “(Trikinetics Inc, Waltham, MA, USA)”  **Response: It is added** | |
| 143-144 | **144-146** | | | I see that this automatic device allows to record much and detailed data simultaneously. However, you studied small groups of flies (from 12-14 up to 32 specimens) each of them obtained from just one cohort. I wonder if you could have obtained more variable data studying flies from different cohorts. Can you provide citations to support the robustness of your choice or there are evidences of intra-group variability highlighted by this kind of study, for instance in Drosophila?  **Response: The number of flies used for the W mated and virgin AR flies was small, and dead flies during the bioassay had to be removed. We have added as reference a work of Chiu et al. [**Chiu, J., Kaub, K., Zou, S. *et al.* Deleterious effect of suboptimal diet on rest-activity cycle in *Anastrepha ludens* manifests itself with age. *Sci Rep* **3**, 1773 (2013). <https://doi.org/10.1038/srep01773>**] with *Anastrepha ludens* flies, where n=16 flies per treatment were used.** | |
| **Results** | | | | | |
| 165-274 | **176-303** | | | In the whole chapter Results (and figure captions as well) mean values are shown followed by “(SE)” without the “±” symbol, moreover standard error values are shown within parentheses after mean numbers. Maybe I am wrong and you use a different syntax I am not familiar with, however I think this way of showing data can confuse the reader. I explain how to show numbers in an example in the following comment.  **Response: The suggested syntax is used throughout the text** | |
| 166-167 | **177-178** | | | I think the sentence should be written as following: “The mean locomotor activity level (±SE) of W virgin males during the LP was 2031.3 ±132.0 counts and that of W virgin females was 1394.2 ± 86.6 counts.” Did you mean this? If yes, please, insert the symbol “±” throughout all the Results chapter and figure captions, before SE values.  **Response: the symbol ± is added throughout the results section were needed** | |
| 166 | **177** | | | Delete “was”.  **Response: deleted** | |
| 185 | **197** | | | Add “during LP” at the end of the sentence, after “females”.  **Response: added** | |
| 187-190 | **202-203** | | | Please, specify that locomotor activity during DP is not shown in Fig 2.  **Response: specified in lines 202-3** | |
| 273-274 | **302-303**  **and Figure 7** | | | In this figure caption you specified colors used in the graph for the two categories. It helps in understanding data, however I suggest to change the series names to avoid confusion: substitute “Light period” with “rest episodes” and “Dark period” into “sleep episodes”. Increase also the font size of all the characters except the Y axis.  **Response: The term “sleep episodes” has been replaced by “rest episodes during the DP” throughout the text and also in figure 7.** | |
| **Discussion** | | | | | |
| 277-278 | **306-307** | | | I think this sentence is a bit imprecise since some factors affecting the locomotor activity are clearly defined in the two options, while other are not. To be more consistent, what about writing “This study indicates that artificial rearing, mating status and photoperiod phase affect the  locomotor activity of the olive fruit fly adults.”  **Response: The suggested phrase was included** | |
| 279 | **308** | | | Change “during this period” into “during this phase”  **Response: changed** | |
| 282 | **310** | | | Change “in accordance to [34].” into “in accordance to past studies [34].”  **Response: changed** | |
| 286 | **-** | | | I do not understand what you mean: how monogamy could cause lower locomotor levels?  **Response:** **The phrase has been removed, as the discussion has been re-written** | |
| 289 | **316-319** | | | When you write “due to oviposition behavior”, do you mean that oviposition might consume energy so that females need longer rest? If yes, write it down more clearly.  **Response: The explanation to our hypothesis is given in lines 316-9**  **We suggest that ovipositing females that do not move inside the tube, thus do not break any beams for several minutes.** | |
| 289-300 | **336-343**  **References 34, 40, 44** | | | Although present findings are compared, discussed and supported by relevant literature, I think that the huge work by Economopoulos has to be acknowledged and taken into consideration. He studied the mating behavior of wild and artificially reared olive fruit flies, evidencing some differences highlighted also by your research, for instance the earlier mating time of the AR males (without mentioning genetic changes between W and AR flies). I am suggesting only some articles that should be discussed: <https://doi.org/10.1093/ee/11.1.17>; Economopoulos, A. P., Zervas, G. A., 1982. The quality problem in olive flies produced for SIT experiments. In: *Sterile insect technique and radiation in insect control*. International Symposium on the Sterile Insect Technique and the use of Radiation in Genetic Insect Control, IAEA SM-255/39: 357-368.  **Response: the discussion has been re—written and references to Economopoulos work and others have been added. See references number 34, 40, 44 and lines 336-343 of the revised MS** | |
| 301 | **325** | | | Delete “of”.  **Response: deleted** | |
| 332 | **-** | | | It seems this sentence refers to the article [50], so, please, insert “[50]” at the end of the sentence.  **Response: This sentence is removed in the revised MS** | |
| 337 | **368-359** | | | Before or after this last paragraph I suggest to recall what stated in the last sentence of the abstract “We discuss how locomotor activity and sleep pattern differences may affect the laboratory flies’ ability to compete with wild males in the field.” You should relate your findings to the importance of producing high quality mass reared insects for SIT purposes.  **Response: A new paragraph had been added, lines 368-359** | |
| **References** | | | | | |
| 31 | **28** | | | Amend “Kouloussis” instead of “Koulousis”  **Corrected** | |
| 40 | **39** | | | Write “Coquillet” instead of “COQUILLET”  **Corrected** | |
| **Figures** | | | | | |
| Figure 1 | | | | Please, increase the font size of the legend (W virgin male etc.)  **Response: font increased** | |
| Figure 2 | | | | Please, increase the font size of all the characters.  **Response: font increased** | |
| Figure 3 | | | | Please, increase the font size of the legend (AR virgin male etc.)  **Response: font increased** | |
| Figure 5 | | | | Please, increase the font size of all the characters except the Y axis  **Response: font increased** | |
| Figure 6 | | | | I suggest to maintain the same pale and dark gray colours used in previous figures. Increase also the font size of all the characters except the Y axis.  **Response: font increased and colors changed** | |
