# Supplementary material for: Artificial diet alters activity and rest patterns in the olive fruit fly
Source: PLoS One. 2023 Feb 21;18(2):e0274586. doi: 10.1371/journal.pone.0274586 (PMC9943008; doi:10.1371/journal.pone.0274586)
Supplement: Attachment — Submitted filename: Response to Reviewers.docx [file pone.0274586.s008.docx]

**Response to Reviewer’s #2 comments:**

Reviewer #2: The revised manuscript was notably changed and improved, mainly thanks to criticism raised by the reviewer 1. All the issues have been addressed. Moreover, I found the additional experiment useful and new observations and comments explain the results more clearly.

According to the new accounts, it seems that flies are not perfectly still during rest episodes since they move their proboscis both in light and dark periods. I understand this is beyond the manuscript’s purpose, however I wonder whether this behavior might be an “involuntary” movement or what else. Was it observed in both sexes? Might it be possibly related to their physiological state?

**Response:** (Line 244 of the Revised Manuscript with track changes) “Protrusion and retraction of the proboscis were observed in both sexes, in otherwise immobile flies.”

We noted that this behavior was observed in both sexes. It could be an involuntary movement of the fly, but as we lack any more data to support this hypothesis, we did not elaborate any further on this topic in the manuscript.
I am listing some very minor revisions, although I recommend to check the revised manuscript for English language since some sentences do not sound correct.

The manuscript was checked by a proficient colleague, mentioned in the Acknowledgment section.

Point to point response to comments

| **Line number** | **Revised MS with Tracked Changes**  **Line number** | **Comments +** responses |
| --- | --- | --- |
| **130** | **111** | edit “Glycine” instead of “Glysine”  Corrected to “Glycine” |
| **168-174** | **153** | specify that wild olive fruit flies have been used.  Response: “Only wild virgin olive fruit flies were used for this bioassay.” |
| **354** | **334** | delete “of”  Corrected |
| **422** | **375** | I think you intended writing “from” instead of “for  Corrected |
| **439** | **391** | Figure S1: Can you specify in the figure caption if the flies are a male and a female? That cannot be clearly evaluated for the fly on the left.  “Resting male (left) and female (right) olive fruit flies during the light period.” |
| **441** | **393** | Please, specify in the figure caption “Resting olive fruit fly female during”  “Resting female olive fruit fly during the dark period” |
| **442** | **394** | Please, specify in the figure caption if the fly is a male or a female  “Resting female olive fruit fly during the light period.” |
| **444** | **396** | Please, specify in the figure caption if the fly is a male or a female  “Lateral view of a resting male olive fruit fly during the dark period.” |
